# Supplementary figures and images for: How tank-mix adjuvant type and concentration influence the contact angle on wheat leaf surface
Source: PeerJ. 2023 Nov 21;11:e16464. doi: 10.7717/peerj.16464 (PMC10668805; doi:10.7717/peerj.16464)

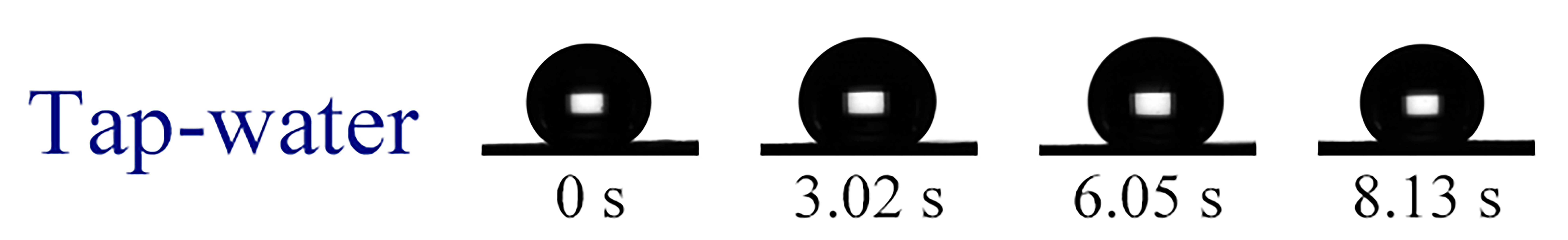

Supplement: Supplemental Information 1 [file peerj-11-16464-s001.png]

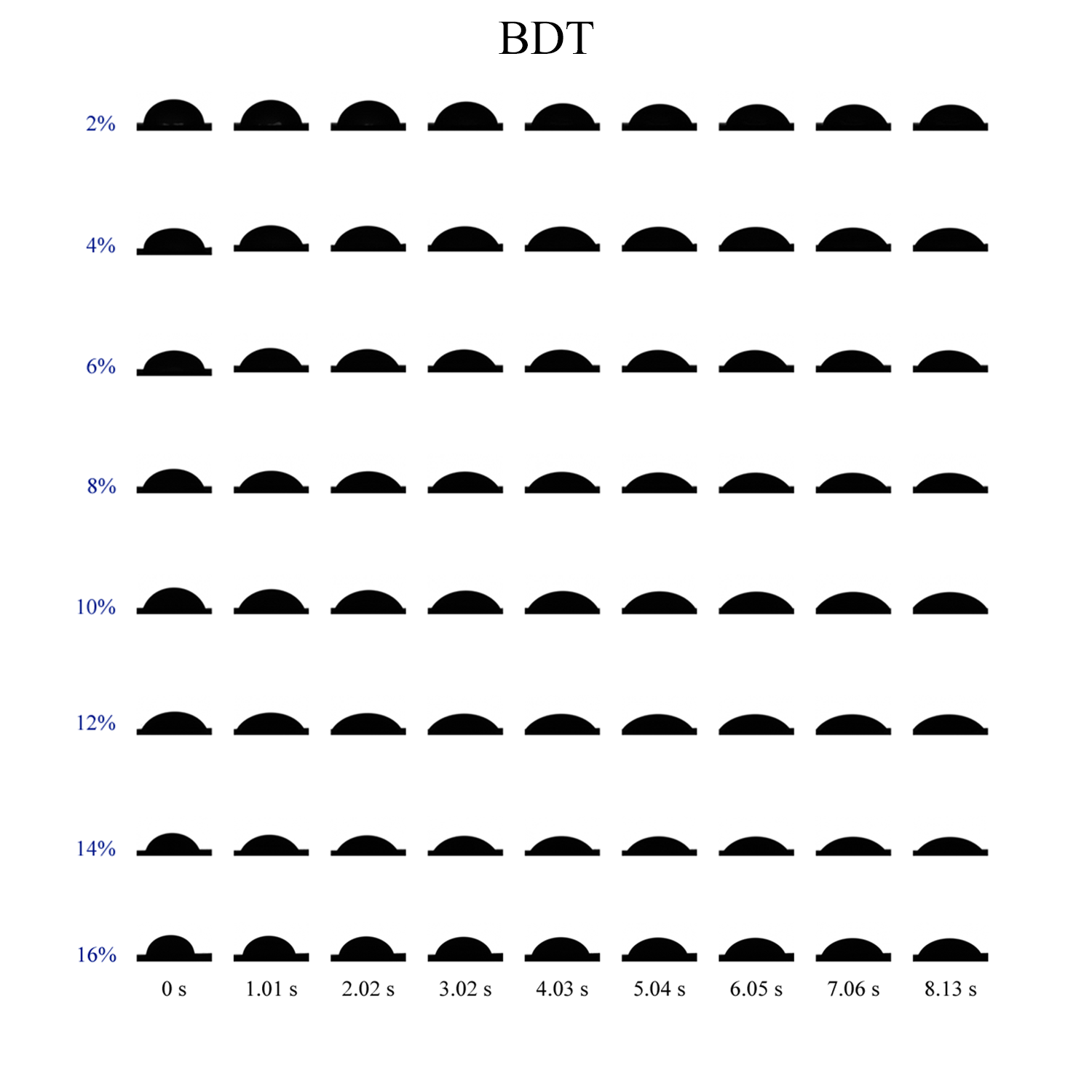

Supplement: Supplemental Information 2 [file peerj-11-16464-s002.png]

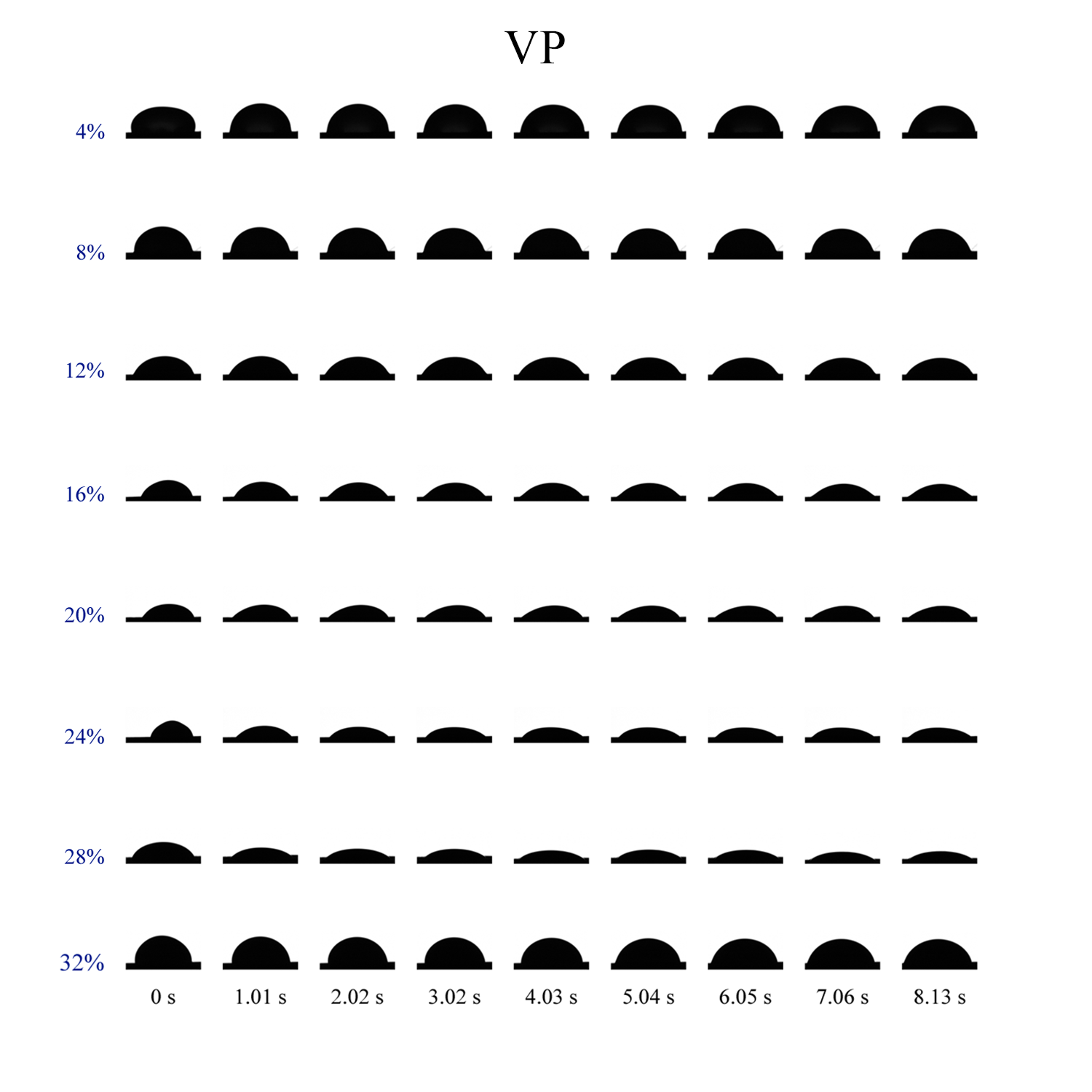

Supplement: Supplemental Information 3 [file peerj-11-16464-s003.png]

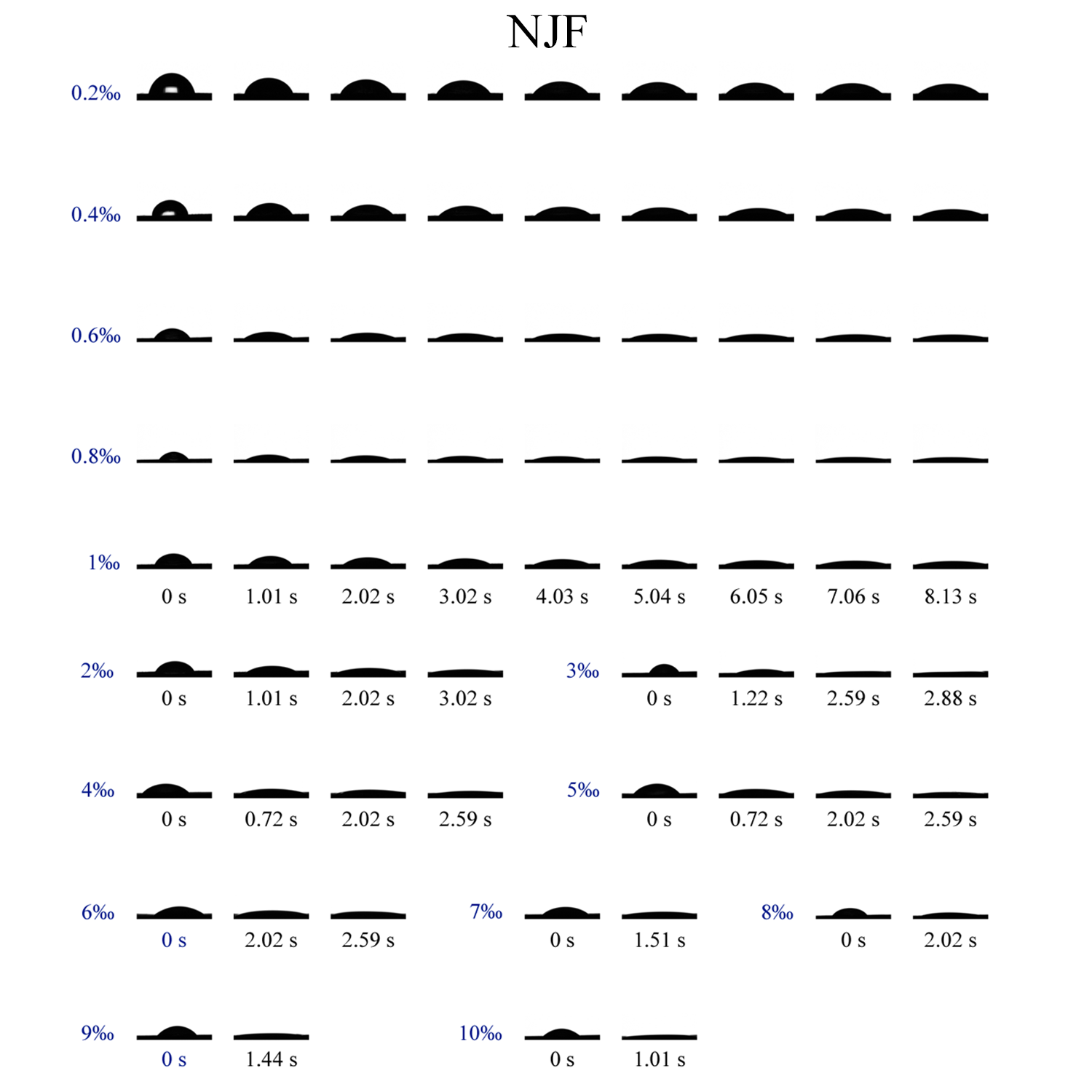

Supplement: Supplemental Information 4 [file peerj-11-16464-s004.png]

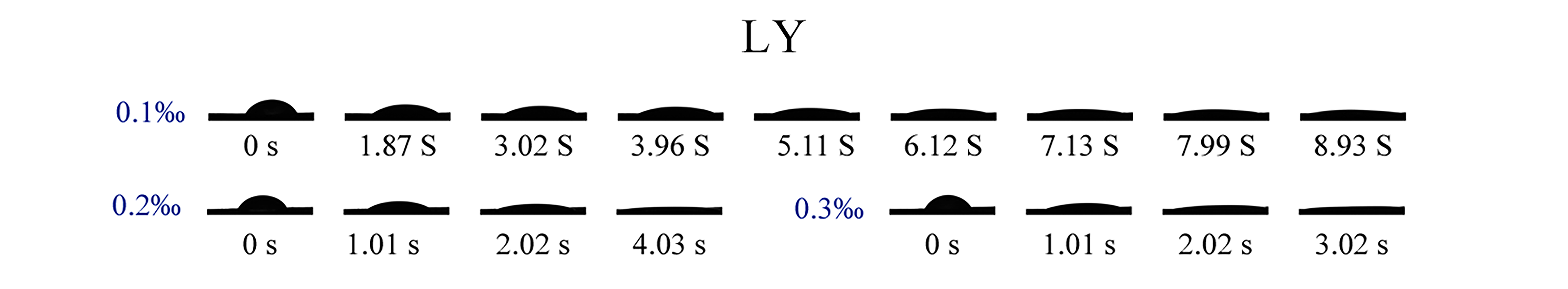

Supplement: Supplemental Information 5 [file peerj-11-16464-s005.png]
